# The appointment of a new Editor-in-Chief

**DOI:** 10.5713/ab.250906

**Published:** 2025-11-25

**Authors:** The Editorial Committee of Animal Bioscience

The Animal Bioscience editorial board has named Prof. Cheol-Heui Yun as the new Editor-in-Chief (EiC), succeeding Prof. Jong Kyu Ha, who has served in this role for 25 years. The term as a new Editor-in-Chief will begin on January 1, 2026.

Prof. Yun, the new EiC, holds his Ph.D. in immune regulation and mucosal immunology from the University of Saskatchewan in Canada. He advanced his professional career at prestigious research institutions worldwide, including the International Vaccine Institute (IVI, Korea), the USDA and NIH (MD, USA), and Gothenburg University (Sweden), where he conducted research on stress, vaccine/adjuvant, infection, and host protective immunity. Professor Yun has been affiliated at Seoul National University since 2006.

Additionally, Prof. Yun has extensive experience in journal publication. He has a lengthy history of working with Animal Bioscience, having served as a reviewer, editorial member, associate editor, and co-editor-in-chief. He serves as the chair of the Committee on Publication Ethics and concurrently has the role of the vice president of the Korean Council of Science Editors. Additionally, he is an ethics editor for both Science Editing and the Korean Journal of Women Health Nursing. He previously served as Secretary General of the Council of Asian Science Editors.

All members of the Animal Bioscience express their hope that this position will contribute to enhancing the journal’s international recognition and advancing economic animal production in both research and industry sectors, particularly in the Asian-Australasian region and beyond.

